# Two dimensional nanosheets as immunoregulator improve HIV vaccine efficacy†

**DOI:** 10.1039/d1sc04044h

**Published:** 2021-12-07

**Authors:** Ye Liu, Yekkuni L. Balachandran, Zulan Li, Yulong Cong, Yiming Shao, Xingyu Jiang

**Affiliations:** Shenzhen Key Laboratory of Smart Healthcare Engineering, Department of Biomedical Engineering, Southern University of Science and Technology No. 1088 Xueyuan Rd, Nanshan District Shenzhen Guangdong 518055 P. R. China jiang@sustech.edu.cn; Institute of Medical Biology, Chinese Academy of Medical Sciences and Peking Union Medical College Kunming Yunnan 650000 P. R. China liuye@imbcams.com.cn; Clinical Laboratory of South Building, Chinese P. L. A. General Hospital No. 28 Fuxing Road Beijing 100853 P. R. China; State Key Laboratory of Infectious Disease Prevention and Control, National Center for AIDS/STD Control and Prevention, Chinese Center for Disease Control and Prevention Beijing P. R. China

## Abstract

Two-dimensional (2D) nanosheets as carriers have shown promising potential for surface-displaying or loading various drugs. Nevertheless, developing sheet-like materials themselves into an immunoregulator has never been realized so far. In this study, we take advantage of the immunoregulatory effects of rare earth elements themselves and develop water-soluble erbium–dysprosium 2D nanosheets (2D NSs). Such 2D NSs can target lymph nodes and activate macrophages to improve vaccine efficacy in mice significantly. Transcriptome analysis further reveals that six critical molecules (Msr1, Ccr2, Serpinb9, Klrk1, Klrd1, Klrc1) closely correlate with 2D NS-mediated immunoregulation *in vivo*. For the first time, the present work realizes a proof-of-concept for designing immunoregulatory 2D NSs and shows a promising potential of 2D NSs for improving the immunoprophylaxis/immunotherapy of vaccines.

## Abstract 2

2D NSs target lymph nodes and activate macrophages to enhance vaccine-induced immune responses *via* regulating six critical genes (Ccr2, Serpinb9, Klrk1, Klrd1, Klrc1, Msr1).

## Graphical abstract

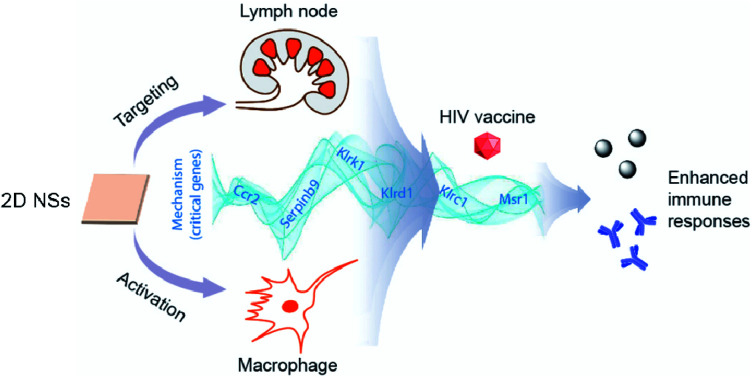

## Introduction

2D materials as carriers have shown their great potential for effectively loading and delivering various drugs *in vivo* due to their unique morphological diversity, structural porosity, and compositional tenability.^1–4^ Nevertheless, developing 2D materials into immunoregulatory drugs has never been realized so far. One of the biggest challenges is introducing applicable immunoregulatory building blocks to endow planar materials with an inherent immunoregulatory capability.^5,6^

Of note, a few rare earth elements or rare earth element-based complexes have shown their unique effects on regulating the functions or behaviors of macrophages, one type of critical immune cell for presenting antigen, eliminating inflammation, and regulating immunity *in vivo*.^7,8^ For instance, erbium (Er) and dysprosium (Dy) can specifically regulate the activation of macrophages,^9–12^ instead of other immune cells such as dendritic cells,^13^*via* neutralizing the reactive oxygen species (ROS) or affecting the production of nitric oxide (NO). Lanthanum (La) can induce bone marrow-derived macrophages to release robust IL-1β to regulate the proliferation, differentiation, and apoptosis of multiple lymphoid cells (T cells, natural killer cells, dendritic cells).^14,15^ Neodymium (Nd) stimulates pattern recognition receptors on the surface of macrophages to regulate intracellular ROS production, which further affects the balance of the immune system.^16^ We, therefore, hypothesize that introducing these rare earth elements as building blocks might endow planar materials themselves with an inherent immunoregulatory capability.

Considering that multiple types of rare-earth elements (*e.g.* Er, Dy, La, Nd) have immunoregulatory effects on the functions or behaviors of immune effector cells, how to select rare-earth elements rationally is a core question for the design of immunoregulatory 2D NSs. In comparison to La and Nd which would cause cellular membrane permeabilization to damage the structure of cells,^12^ as well as over-induce inflammasome activation to disturb the balance of Ca^2+^ influx and cause cell death,^16^ Er and Dy exhibit better biosafety *in vivo*.^12,17,18^ Moreover, employed Er and Dy 2D NSs can more comprehensively use the immunoregulation functions of both Er (ROS-based mechanism)^10^ and Dy (NO-based mechanism),^11^ in comparison with either Er- or Dy-alone 2D NSs.

In the current study, we design and synthesize a new type of 2D erbium–dysprosium nanosheets with good water solubility and satisfactory biosafety *in vivo*. We use such 2D NSs as an immunoregulator to significantly enhance humoral and cellular responses induced by the HIV vaccine in mice. We further explore multiple critical genes, which can regulate 2D NSs-mediated immunoregulation *in vivo*. These results provide insights into the mechanism of such a new immunoregulator and better guide us on optimizing the design of other 2D materials with immunoregulatory effects. Overall, our study realizes a proof-of-concept for developing new immunoregulatory materials and shows promising potential for using artificially constructed 2D materials to improve vaccine-induced immunities.

## Results and discussion

### Characterization of 2D NSs

**Fig. 1:**
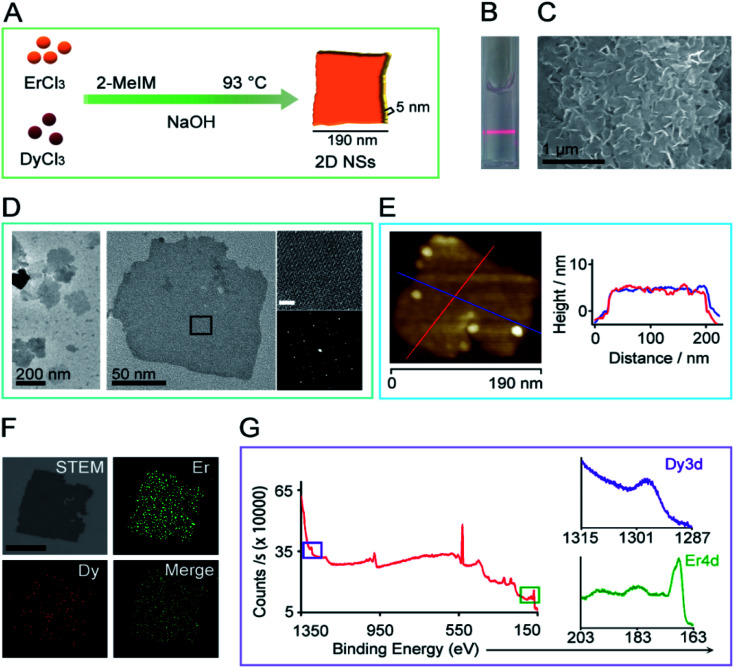
The synthesis and characterization of 2D NSs. (A) The synthesis scheme of 2D NSs. (B) Laser scattering of well-dispersed 2D NSs solution due to Tyndall effect. (C) Sheet-like morphology was observed under SEM of samples dispersed on a silicon wafer. (D) TEM image of 2D NSs formed at a ratio of 7 : 1 of Er ions to Dy ions. Enlarged lattice-resolved aberration-corrected HRTEM image taken from the marked area. Below shows the corresponding fast Fourier transform/FFT pattern (scale 2 nm). (E) AFM detects the height of 2D NSs. A red and blue line across a single 2D NSs is drawn in two different directions to collect the height of 2D NSs. (F) Dark-field STEM image and EDS mapping show the distribution of Er and Dy throughout 2D NSs. Scale in STEM is 100 nm. (G) XPS analysis of the binding energy of Er and Dy element in 2D NSs.

An alkaline aqueous solution of erbium chloride, dysprosium chloride, and 2-methylimidazole was incubated at 93 °C for 48 hours to form 2D NSs. We have tried a series of the ratio of Er ions to Dy ions (8 : 0, 7.9 : 0.1, 7.5 : 0.5, 7 : 1), and obtained the size-controlled 2D NSs only at a ratio of 7 : 1 of Er : Dy (Fig. 1A, ESI Fig. S1†). The 2D NSs appear finely suspended, as shown by the Tyndall scattering effect of the 2D NSs solution (Fig. 1B). Fig. 1C shows a typical scanning electron microscopy (SEM) image of the 2D NSs deposited on a silicon wafer. Transmission electron microscopy (TEM) shows the finely dispersed sheet-like morphology measuring edge length less than 200 nm (measured along the longest edges of NSs) (Fig. 1D). The left in Fig. 1D is the aberration-corrected high-resolution TEM (JEM-ARMF200) image and the corresponding FFT (below) validating the single crystallinity of the 2D NSs. Atomic force microscopy (AFM) image of single 2D NSs detected the uniform thickness of ∼5 nm in either direction marked with different color lines (Fig. 1E). Energy dispersive spectroscopy (EDS) mapping showed the chemical makeup of 2D NSs. The distribution of Er and Dy elements are homogeneous throughout the sheet (Fig. 1F, ESI Fig. S2†) and the ICP-MS measured 78% of Er and 22% of Dy elements (ESI Fig. S3†). Furthermore, X-ray photon spectroscopy (XPS) analysis confirmed the presence of Er and Dy elements with binding energies at 169 eV (Er4d) and 1297 eV (Dy3d) (Fig. 1G). To study the fine structure we obtained the spectra using AlKα X-ray source. The binding energy (BE) scale is corrected with reference to carbon 1s peak at BE of 284.5 eV. The Dy 3d5/2 spectra exhibited a single component at BE around 1299 eV, which correspond to metallic Dy (Dy^0^) chemical state (ESI Fig. S4A†). The Er 4d5/2 spectra exhibited two components at BEs around 170 and 168 eV. The former component is related to the metallic Er (Er^0^) chemical state and the latter to Er-oxide (Er^3+^), respectively (ESI Fig. S4B†).^19^ The presence of the oxide component is due to surface oxidation of the Er/Dy NSs. The oxidation is predominant in Er due to the larger composition of Er in the Er/Dy NSs. 2D NSs can maintain their morphology and zeta value (+27.4 mV) in an aqueous solution for more than one month at 4 °C (ESI Fig. S5†).

### 2D NSs on regulating the function of macrophages

**Fig. 2:**
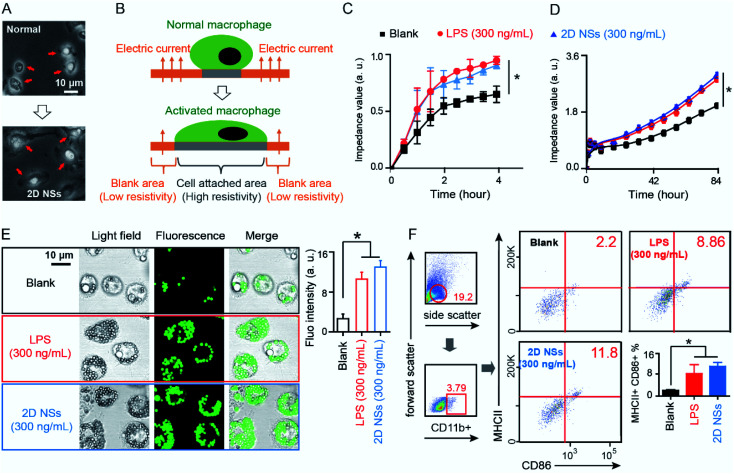
The activation of macrophages by 2D NSs. (A) An optical microscope observes the morphology of macrophages after stimulating with/without 300 ng mL^−1^ 2D NSs for three days. (B) The scheme shows activated macrophages extend their size to increase the electrical resistivity of the cell culture plate. (C) The change of electrical resistivity of the macrophage-cultured plate during 4 hours. (D) The change of electrical resistivity of the macrophage-cultured plate during 84 hours. (E) The phagocytosis capability of macrophages treated with/without 2D NSs (300 ng mL^−1^) or LPS (300 ng mL^−1^) at Day 2. (F) Flow cytometry analyze the expression of activated marker (CD86) and antigen-presenting indicator (MHC II) on the surface of macrophages isolated from mice injected with 2D NSs (300 ng mL^−1^) or LPS (300 ng mL^−1^). All the results are shown as the mean value ± SD. * means *p* < 0.05.

We investigated the effect of 2D NSs on regulating the function or behavior of macrophages. We isolated primary macrophages from mouse enterocoelia after three days of a 2 mL paroline injection^20^ and co-cultured 1 × 10^6^ primary macrophages with 2D NSs with different concentration (30 μg mL^−1^, 3 μg mL, 300 ng mL^−1^ and 30 ng mL^−1^). 300 ng mL^−1^ is an optimized concentration for the activation of macrophages (ESI Fig. S6†). Activated macrophages will attach closer to their growth basement and have an obvious extension of cellular morphology (bigger size, pseudopods).^21^ In a visual field, we use an optical microscope to observe that macrophages stimulated with 300 ng mL^−1^ 2D NSs extended their size and grow many pseudopods, which are two critical characteristics to evaluate the activation of primary macrophages according to previous studies.^22^ In contrast, naked macrophages have smaller sizes and exhibit the spherical shape that is a typical half-attachment growth situation of cells (Fig. 2A). We evaluated the activation of macrophages *via* real-time monitoring of the electrical resistivity of the macrophage-cultured plate. As shown in Fig. 2B, in comparison to normal macrophages, activated macrophages will extend their size and cover the broader area of the plate, causing increased electrical resistivity. During 4 hours, macrophages stimulated with 300 ng mL^−1^ 2D NSs significantly increased the electrical resistivity of the plate compared to naked macrophages. A similar increase of electrical resistivity appears in the plate cultured with macrophages stimulated with 300 ng mL^−1^ lipopolysaccharide (LPS), a positive stimulator for macrophage activation (Fig. 2C).^23^ We observed a continuous increase of electrical resistivity even up to 84 hours (Fig. 2D). All these results solidly supported that 2D NSs can induce the activation of macrophages.

The characteristics of our 2D NSs on activating macrophages might suggest its potential as a general immunoregulator for the enhancement of vaccine efficacy. Among various strategies for enhancing vaccine-triggered immune responses, activating macrophages to improve the uptake and presentation of antigens is a very effective means for improving vaccine efficacy.^24^ Macrophages participate in almost all checkpoint events during the whole process of vaccine-induced immune responses, including the uptake of vaccines, the process of antigens, the presentation of epitopes, and the regulation of the functions of immune effector cells (*e.g.* T cells, B cells).^7^

We evaluated the phagocytosis of 2D NSs-activated macrophages against green fluorescent microspheres. In this test, we selected mouse-derived RAW 264.7 macrophages as a standard cell line, which has been domesticated and widely used to evaluate the functions of macrophages.^25^ At Day 2, 4, 6, and 8, macrophages stimulated with either 300 ng mL^−1^ 2D NSs or 300 ng mL^−1^ LPS produced significantly stronger fluorescence intensity (around 3–4 fold) than naked macrophages (Fig. 2E, ESI Table S1†). These results indicated that 2D NSs-activated macrophages enhanced their phagocytosis capability. Furthermore, we evaluated the activation of *in vivo* macrophages *via* quantifying the surface marker (CD86) on macrophages in mice.^23^ As shown in left part in Fig. 2F, we first gated the living splenocytes (19.2%) in all splenocytes. We further gated CD11b-positive cells (3.79%) as macrophages in all living splenocytes for analyzing the activation of macrophages. Flow cytometry analysis revealed that 300 ng mL^−1^ 2D NSs significantly increase the expression of CD86 on the surface of macrophages *in vivo*. It is consistent with the result from macrophages stimulated with 300 ng mL^−1^ LPS (Fig. 2F). Importantly, we observed a significantly increased expression of MHC II marker for antigen presentation^26^ on the surface of macrophage after stimulating with 300 ng mL^−1^ 2D NSs *in vivo* (Fig. 2F). Furthermore, we also evaluate macrophage-derived production of reactive oxygen species (ROS) and nitric oxide (NO) induced by 2D NSs. 300 ng mL^−1^ 2D NSs can facilitate macrophages to significantly enhance the expression of both ROS (ESI Fig. S7†) and NO (ESI Fig. S8†). Together, these results indicated that 2D NSs activated macrophages to enhance their phagocytosis capability and bioactivity for the increased presentation of antigens.

### 2D NSs targeting lymph node

**Fig. 3:**
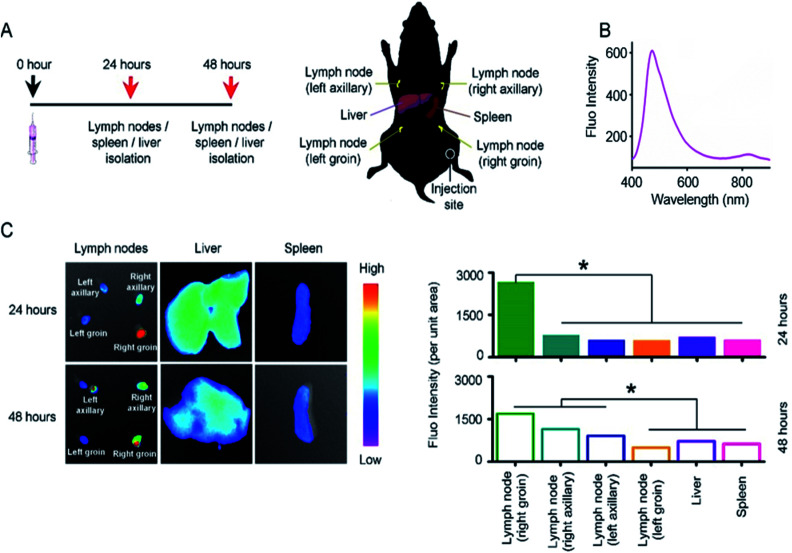
Lymph node targeting of 2D NSs *in vivo*. (A) Scheme: we injected 30 ng 2D NSs into each mouse. At the 24 and 48 hours, we surgically removed the lymph nodes at four different positions (left axillary, right axillary, left groin, right groin), spleen and liver. (B) The fluorescence emission spectrum of 2D NSs. (C) The fluorescence images (left part) and fluorescence intensity (right part) of lymph nodes, spleen, and liver from 2D NSs-injected mice. All the results are shown as the mean value ± SD. * means *p* < 0.05.

We investigated the targeting behavior of 2D NSs *in vivo*. We can track the targeting behavior of 2D NSs *via* detecting Er/Dy-derived fluorescence signals.^27^ Moreover, the lymph node and spleen are the most important organs/tissues for mediating immunity *in vivo*.^28,29^ The liver is the critical organ, which is in charge of the metabolism of nanomaterials *in vivo*.^30^ We, therefore, observed the targeting of 2D NSs to these organs/tissues. We injected 30 ng 2D NSs into each mouse. At the 24 and 48 hours, we harvested lymph nodes at four different positions (left axillary, right axillary, left groin, right groin), spleen, and liver from 2D NSs-injected mice (Fig. 3A). Significantly higher fluorescence intensity (2D NSs-derived fluorescence, Fig. 3B) appeared in lymph nodes at the 24 and 48 hours, in comparison with other organs (*p* < 0.05, Fig. 3C). These results proved that 2D NSs targets lymph nodes *in vivo*. Considering lymph node is a critical type of immunological tissue for mediating vaccine-induced immune responses,^28^ such lymph node targeting suggested that 2D NSs as an immunoregulator might improve the efficacy of other vaccines effectively.

Of note, 2D NSs carry positive zeta potentials (+27.4 mV, ESI Fig. S4†) in an aqueous solution, and the surface charge of DNA vaccines is negative. For the co-administration of 2D NSs and DNA vaccines *in vivo*, 2D NSs can adsorb DNA vaccines by electrostatic attraction (ESI Fig. S9†) and deliver these DNA vaccines to lymph nodes. A similar phenomenon also is commonly observed between DNA molecules and other 2D nanosheets, such as graphene.^31^ Therefore, 2D NSs in the current study may play a dual-functional role in both activating immune cells and target delivering DNA vaccines to lymph nodes.

### 2D NSs facilitated immune responses HIV DNA vaccine

**Fig. 4:**
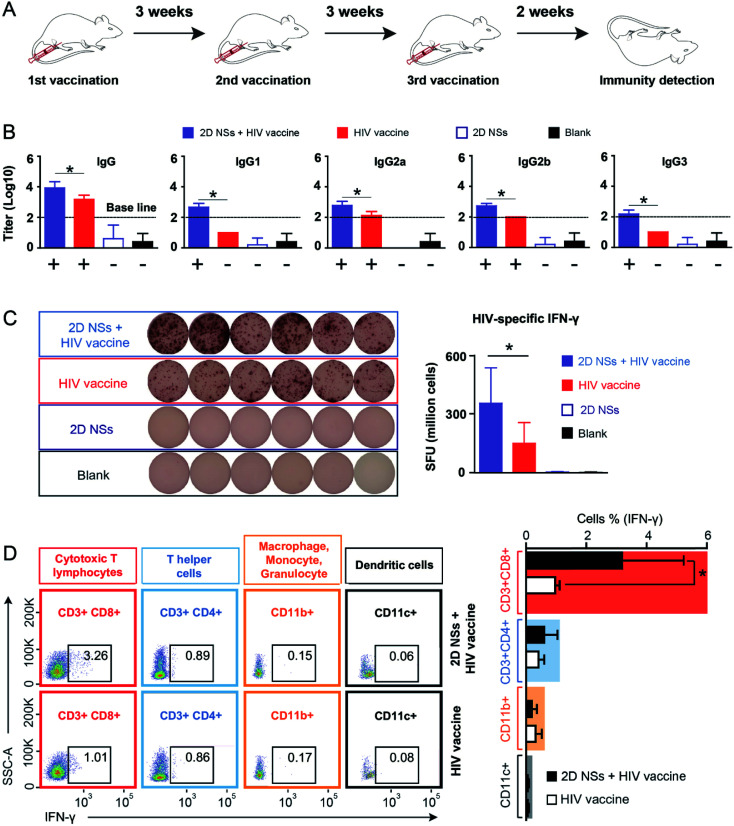
The effect of 2D NSs on regulating HIV DNA vaccine-triggered immune responses. (A) The scheme of vaccination: Mice (six mice per group) receive three vaccinations. The interval between two vaccinations is three weeks. Fresh splenocytes and serums are harvested 15 days after the final immunization. (B) Enzyme-linked immunosorbent assay/ELISA : HIV DNA vaccine-triggered antibody response (IgG and IgG subclasses) regulated by 2D NSs in mice. We define the positive IgG/IgG subclass response (over baseline) as that the OD value (*Δ* value between 450 nm and 630 nm) is no lower than 0.1 when serum is diluted at 1 : 100 (log 10 value is no lower than 2). + and − means positive and negative antibody response. (C) Enzyme-linked immunospot assay/ELISPOT:HIV DNA vaccine-triggered T cell response (IFN-γ) regulated by 2D NSs in mice. More than 20 counting in 1 × 10^6^ splenocytes is considered positive. (D) Flow cytometry analysis: the production of HIV-specific IFN-γ from different types of immune cells (cytotoxic T lymphocyte/CD3^+^CD8^+^, T helper cells/CD3^+^CD4^+^, macrophage/monocyte/granulocyte/CD11b^+^, dendritic cell/CD11c^+^) regulated by 2D NSs in mice. All the results are shown as the mean value ± SD. * means *p* < 0.05.

Based on our finding that 2D NSs can activate macrophages to enhance the uptake and presentation of antigens, we further investigated whether 2D NSs can facilitate the HIV DNA vaccine to trigger stronger immune responses in the mouse model (Fig. 4A). We use DNA vaccine candidates to evaluate the immunoregulation of our 2D NSs. DNA vaccines highlight the advantages of precise construction, easy production, convenient transport, and satisfactory biosafety.^32^ However, it suffers modest immunogenicity *in vivo*, which greatly limits its practical application.^32,33^ Enhancing DNA vaccine-triggered immune responses, therefore, becomes a long-sought aim of many immunologists. The use of our 2D NSs simultaneously enhances both humoral and cellular immune responses induced by the HIV DNA vaccine *in vivo*. Such a balanced enhancement of HIV-specific humoral and cellular immune responses regulated by 2D NSs might be an unparalleled advantage for realizing the neutralization (mediated by humoral response) and cytotoxicity (cellular response) against HIV, in comparison with other most of the reported immunoregulators, which only can enhance either humoral or cellular responses induced by HIV vaccines.^34–41^

For the antibody response, we quantified the titer of HIV-specific IgG and its four subclasses (IgG1, IgG2a, IgG2b and IgG3) regulated by 2D NSs *in vivo*. HIV-specific IgG titer increased around 4 fold in mice vaccinated with 20 μg HIV DNA vaccine with 30 ng 2D NSs, compared to that vaccinated with 20 μg HIV DNA vaccine alone (Fig. 4B). We further analyzed which IgG subclasses majorly contribute to 2D NSs-caused enhancement of IgG response. Naked HIV DNA vaccine-induced two HIV-specific IgG subclass responses (positive IgG2a and IgG2b responses) in mice. By contrast, the introduction of 2D NSs allowed the HIV DNA vaccine not only to enhance the titer of IgG subclasses (*p* < 0.05) but also to broaden IgG subclass response (positive IgG1, IgG2a, IgG2b and IgG3 responses) (Fig. 4B). Notably, a broad IgG subclass response appears in HIV long-term non-progressors infected by HIV but does not or very slowly develop into AIDS.^42–45^ It suggests that 2D NSs might help HIV vaccine to inhibit the progress of HIV better. Of course, more solid data is necessary to confirm such a hypothesis. For T cell response, we evaluated the production of HIV-specific IFN-γ from immune effector cells in mice. The expression of HIV-specific IFN-γ significantly enhanced (2.3 fold) in mice vaccinated with 20 μg HIV DNA vaccine with 30 ng 2D NSs, compared to that vaccinated with 20 μg HIV DNA vaccine alone (Fig. 4C). This result demonstrated that 2D NSs facilitated the HIV DNA vaccine to induce a stronger T cell response in mice. Moreover, we analyzed the source of HIV-specific IFN-γ to understand the immunoregulation effect of 2D NSs on the HIV DNA vaccine. We selected multiple subgroups of immune cells, including macrophages/monocytes/granulocytes, cytotoxic T lymphocytes, T helper cells, and dendritic cells^46^ because these immune cells majorly produce IFN-γ *in vivo*.^47,48^ For the naked HIV DNA vaccine, both cytotoxic T lymphocytes (1.01%) and T helper cells (0.86%) produced most of HIV-specific IFN-γ (Fig. 4D). A much smaller part of HIV-specific IFN-γ derived from macrophages/monocytes/granulocytes (0.17%) and dendritic cells (0.08%). Although the introduction of 2D NSs failed to induce more T helper cells (0.89%), macrophages/monocytes/granulocytes (0.15%), and dendritic cells (0.06%) to produce HIV-specific IFN-γ, it significantly enhanced cytotoxic T lymphocytes (from 1.01% to 3.26%, *p* < 0.05) to produce HIV-specific IFN-γ (Fig. 4D). These results indicated that 2D NSs majorly regulated cytotoxic T lymphocytes to enhance the expression of HIV-specific IFN-γ in mice. Overall, 2D NSs as immunomodulators can significantly improve both antibody and T cell response induced by HIV DNA vaccine in mice.

### Molecular mechanism of 2D NSs improving HIV DNA vaccine

**Fig. 5:**
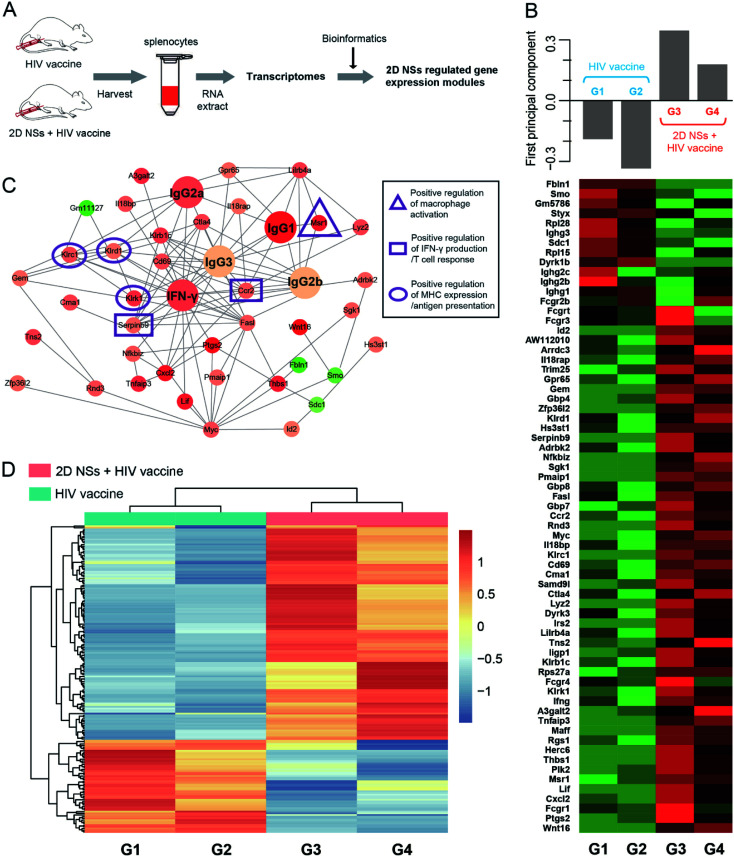
Transcriptome profiling in mice treated with HIV vaccine alone or HIV vaccine + 2D NSs. (A) The diagram shows the procedures to characterize the transcriptomes of the specific spleen in mice treated with HIV DNA vaccine or HIV DNA vaccine + 2D NSs. The dose of HIV DNA vaccine and 2D NSs is 20 μg and 30 ng. (B) Protein–protein interaction (PPI) analysis based on STRING database identifies highly correlated gene modules. We show the top 67 most variable transcripts *via* principal component analysis. (C) Based on the top 100 genes from the immune-related module, we show the extensive topographical overlap among their expression patterns, especially for macrophage activation, T cell response/IFN-γ production, and antigen presentation. (D) Hierarchical clustering analyses of differentially expressed genes (DEGs) from mice treated with HIV vaccine alone or HIV vaccine + 2D NSs.

To understand the molecular mechanism of 2D NSs improving HIV DNA vaccine-triggered immune responses *in vivo*, we analyzed the transcriptomes^49^ of the splenocytes isolated from the mice treated with 30 ng 2D NSs (Fig. 5A). We employed protein–protein interaction (PPI) analysis based on the STRING database to identify the highly correlated gene modules, which have significantly up-regulated or down-regulated expression (Fig. 5B). Principal component analyses of the top 67 most variable transcripts revealed a significant up-regulation of immunomodulatory gene module in 2D NSs treated mice (Fig. 5B). Cluster Profiler gene ontology enrichment analyses of gene module revealed that six genes are at the center of this interconnected network (Msr1, Ccr2, Serpinb9, Klrk1, Klrd1, and Klrc1) showed the most related with 2D NSs-regulated HIV vaccine-triggered immune responses *in vivo* (Fig. 5C). According to previous reports, three natural killer cell lectin-like receptor subfamily genes (Klrk1, Klrd1, and Klrc1)^50^ will effectively enhance the presentation of antigens. Such enhancement is possibly caused by activating critical antigen-presenting cells (APCs), such as dendritic cells and macrophages. Further investigation also found a significantly up-regulated expression of Msr1, a critical gene to activate macrophages.^51^ Moreover, two other genes (Ccr2 and Serpinb9) that play critical roles in up-regulating cytokine production^52,53^ showed increased expression, which is consistent with the activation of macrophages. The results mentioned above showed an immunoregulation-related network involving the activation of immune cells, antigen presentation, and the production of immune effectors. Such an immunoregulatory network might explore why 2D NSs can facilitate the HIV DNA vaccine to trigger stronger immune responses. Moreover, hierarchical clustering analyses of differentially expressed genes (DEGs) (*p*-value < 0.05) confirmed that the significantly different transcriptional profiles between 2D NSs and HIV DNA vaccine-treated mice and HIV DNA vaccine-treated mice (Fig. 5D). Together, transcriptomes data revealed that 2D NSs could regulate Msr1, Ccr2, Serpinb9, Klrk1, Klrd1 and Klrc1 gene to enhance HIV DNA vaccine-triggered immune responses *in vivo*.

### *In vitro* and *in vivo* biosafety of 2D NSs

**Fig. 6:**
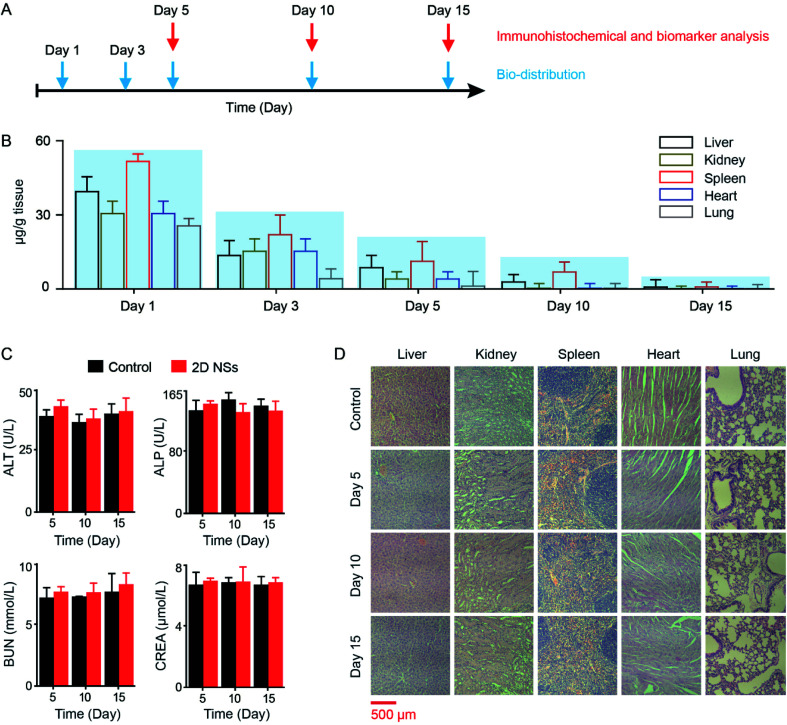
The distribution and biosafety of 2D NSs *in vivo*. (A) Schedule: the distribution and biosafety analysis of 2D NSs *in vivo*. (B) The distribution analysis of 2D NSs in different organs (liver, kidney, spleen, heart, lung). 2D NSs in the liver, kidney, spleen, heart, and lung are quantified by ICP-MS on Day 1, 3, 5, 10, and 15. (C) Four biomarkers (alanine aminotransferase/ALT, alkaline phosphatase/ALP, urea nitrogen/BUN, creatinine/CREA) in serum are detected for evaluating the influence of 2D NSs for the function of the liver (ALT, ALP) and kidney (BUN, CREA). (D) The immunohistochemical analysis of organs (liver, kidney, spleen, heart, lung) at different time points (Day 5, Day 10, Day 15) is used for evaluating the organ/tissue toxicity of 2D NSs. The scale bar is 500 μm. The results are shown as the mean value ± SD.

We studied the biodistribution, cytotoxicity, and *in vivo* toxicity of 2D NSs. We assessed the biodistribution of 2D NSs in five organs (liver, kidney, spleen, heart and lung) at different time points (Day 1, Day 3, Day 5, Day 10, and Day 15) using an inductively coupled plasma-mass spectrometry (ICP-MS) system (Fig. 6A). The residual amount of 2D NSs significantly reduced in mice from Day 1 to Day 15. On Day 15, 2D NSs in the liver, kidney, spleen, heart, and lung have been almost removed (Fig. 6B). We evaluated the biosafety of 2D NSs. 2D NSs exhibited satisfactory cytotoxicity *in vitro*. The viability of HeLa and HUVEC cells has not been significantly reduced after incubation of 2D NSs with a broad range of concentrations (0–300 μg mL^−1^) for 24 hours (ESI Fig. S10†). For the evaluation of *in vivo* biosafety of 2D NSs, we monitored the expression of multiple important biochemical indicators, including alanine aminotransferase (ALT), alkaline phosphatase (ALP), creatinine (CREA), and urea nitrogen (BUN) in serum from 2D NSs-injected mice for 15 days. All four indicators showed no significant changes in 2D NSs-treated mice at Day 5, Day 10, and Day 15, compared to normal mice (Fig. 6C). Furthermore, we performed the histopathologic analysis of multiple organs (heart, liver, spleen, lung, and kidney). There was no evidence to show the infiltration and necrosis of inflammatory cells in these organs from 2D NSs-injected mice. There was no visible difference between 2D NSs-treated mice and normal mice (Fig. 6D). These results prove satisfactory biosafety of 2D NSs *in vitro* and *in vivo*.

## Conclusions

In conclusion, we developed water-soluble 2D NSs into a new immunoregulator. Such 2D NSs can target the lymph node and effectively activate macrophages to improve HIV DNA vaccine-induced immune responses *in vivo* significantly. Molecular mechanism investigation revealed that six genes (Msr1, Ccr2, Serpinb9, Klrk1, Klrd1, and Klrc1) play critical roles in mediating 2D NSs-mediated immunoregulation. The realization of the concept of 2D NSs immunoregulator dramatically broadens the scope for choices to optimize the vaccination of infectious diseases, tumor immunotherapy, and other immune-based prophylaxis and therapy.

## Ethical statement

Animal studies were approved by the Animal Ethics Committee of the Institute of Medical Biology, Chinese Academy of Medical Sciences and Peking Union Medical College and executed according to guidelines from the Committee of Welfare and Ethics of Laboratory Animals in Kunming.

## Data availability

We provide all data in ESI.† All readers can contact the corresponding authors for further discussions.

## Author contributions

Y. L., Y. L. B., and X. J. conceived the project and designed the experiments. Y. L., Y. L. B. and Z. L. performed the experiments and analyzed the data. X. J., Y. C., and Y. S. supervised the study. Y. L., Y. L. B., Z. L., Y. C., Y. S., and X. J. discussed the data and wrote the paper.

## Conflicts of interest

There are no conflicts to declare.

## Supplementary Material

SC-013-D1SC04044H-s001
