# Supplementary material for: Two dimensional nanosheets as immunoregulator improve HIV vaccine efficacy
Source: Chem Sci. 2021 Dec 7;13(1):178–87. doi: 10.1039/d1sc04044h (PMC8694375; doi:10.1039/d1sc04044h)
Supplement: SC-013-D1SC04044H-s001 [file SC-013-D1SC04044H-s001.pdf]

Electronic Supplementary Material (ESI) for Chemical Science.
This journal is © The Royal Society of Chemistry 2021

Supporting Information

Two Dimensional Nanosheets as Immunoregulator Improves HIV Vaccine Efficacy

Ye Liu, [2,†,*] Yekkuni L. Balachandran, [1,†] Zulan Li, [3,†] Yulong Cong, [3] Yiming Shao, [4] Xingyu Jiang [1,*]

[1] Shenzhen Key Laboratory of Smart Healthcare Engineering, Department of Biomedical Engineering, Southern University of Science and Technology, No. 1088 Xueyuan Rd, Nanshan District, Shenzhen, Guangdong 518055, P. R. China.

[2] Institute of Medical Biology, Chinese Academy of Medical Sciences and Peking Union Medical College, Kunming, Yunnan, 650000, P. R. China.

[3] Clinical Laboratory of South Building, Chinese P. L. A. General Hospital, No. 28 Fuxing Road, Beijing 100853, China.

[4] State Key Laboratory of Infectious Disease Prevention and Control, National Center for AIDS/STD Control and Prevention, Chinese Center for Disease Control and Prevention, Beijing, China.

* Corresponding authors. Email: jiang@sustech.edu.cn, liuye@imbcams.com.cn.

† The authors equally contribute this work.

Materials and Methods

Materials

Dysprosium chloride (III) hexahydrate and Erbium chloride (III) hexahydrate are from Strem Chemicals LLC. Sodium hydroxide and 2-methylimidazole are from TCI Chemicals LLC. Other chemical reagents are from Sigma-Aldrich LLC.

Mice

Female Bal B/C mice (6−8 weeks old) are obtained from the Institute of Medical Biology, Chinese Academy of Medical Sciences and Peking Union Medical College. Animal studies were approved by the Animal Ethics Committee of the Institute of Medical Biology, Chinese Academy of Medical Sciences and Peking Union Medical College and executed according to guidelines from the Committee of Welfare and Ethics of Laboratory Animals in Kunming.

Synthesis of 2D NSs

After rapidly mixing 2.5 mL erbium chloride solution (35 mg) and 2.5 mL dysprosium chloride (5 mg), 5 mL 2-methylimidazole (160 mg) is added drop by drop. 5 minutes later, the pH of the mixture is regulated to pH 11.5 using freshly-prepared NaOH solution. The whole mixture is transferred into a hot air oven maintained at 93°C for 48 hours. The final product is collected by centrifugation and purified through dialysis for 24 hours. The resultant solution is concentrated and stored at 4°C.

Characterization of 2D NSs

The morphology of 2D NSs is observed by Tecnai G2 20 S-TWIN transmission electron microscopy. The distribution of Er and Dy throughout 2D NSs is analyzed using an energy dispersive spectrum (EDS, Jeol JEM2001 STEM). The crystal structure of 2D NSs is obtained by high-resolution transmission electron microscopy (HRTEM, Jeol JEM2001 STEM). The height of 2D NSs is detected *via* an atomic force microscope (AFM, Bruker dimension icon). The binding energy of Er and Dy element in 2D NSs is analyzed using XPS spectra (Thermo Scientific ESCALAB 250XI). The zeta potential of 2D NSs is obtained using Marlvern zetasizer. The inductively coupled plasma-Mass spectroscopy (ICP-MS) measurements were performed on a PerkinElmer NexION 300X ICP-MS.

Macrophage isolation

Peritoneal macrophages are harvested 72 hours after an intraperitoneal injection of 2 mL paroline per mouse (Bal B/C). 1×10^6 macrophages are seeded in a cell-cultured plate (NUCE) with DMEM medium (Hyclone) with 10% FBS (GIBCO) and 1% pen/strep at 37°C and 5% CO_2.

The detection of the electrical resistivity of macrophage-cultured plate

Isolated macrophages (1×10^6 cells/mL) are cultured on the plate, which can conduct electricity. Either a final concentration of 300 ng/mL 2D NSs or 300 ng/mL lipopolysaccharide (LPS) is added into each well to stimulate macrophages. The

electrical resistivity of the plate is real-time monitored using real-time cellular analysis (RTCA S16, ACEA Biosciences. LLC) from 0 to 84 hours. Macrophages cultured with full cell cultured medium (10% FBS) are used as control.

Reactive oxygen species (ROS) detection

Macrophages were cultured in a T75 cell flask. The cells were transferred into 24-well plates with a cell density of 5×10^5 cells/mL. 300 ng/mL 2D nanosheets were co-cultured with the cells for 24 h. Macrophages cultured into the naked medium were reserved as blank control. 10 μmol/L DCFH-DA (Sorlarbio ROS fluorescence detection kit) were added to each well. The cells were incubated at 37°C for 20 min and washed with serum-free cell culture solution three times. The intensity of green fluorescence from each well was detected in real-time by using a fluorimeter (BioTek fluorimeter) at the excitation wavelength of 488 nm and emission wavelength of 525 nm.

Nitric oxide (NO) detection

Macrophages were cultured in a T75 cell flask. The cells were transferred into 24-well plates with a cell density of 5×10^5 cells/mL. 300 ng/mL 2D nanosheets were co-cultured with the cells for 24 h. Macrophages cultured into the naked medium were reserved as blank control. The cells cultured in the plate were digested with trypsin and transferred to EP tubes. The cells were centrifuged at 1000 rpm for 5 min, and the supernatant was discarded. By adding 100 μL extract into each tube and resuscitating the cells with a pipette. The cells were crushed by ice bath ultrasound (power 300 W, ultrasound 3s,

interval 7 seconds total time 3 minutes), and centrifuged at 4°C with 12000 rpm for 15 min. The precipitation was discarded, and the supernatant was taken and placed on ice for testing. We use the SorLARBio NO Test Kit to detect NO. We added either 20 μL reagent 1 or 20 μL distilled water into each tube. We added 20 μL reagent II into each tube at room temperature for keeping 5 min, centrifuged with 3500 rpm at 4°C for 10 min. By adding 100 μL supernatant and chromogenic solution to each well, we measured the absorbance value at 550 nm.

Mice vaccination

Mice are randomly divided into four different groups (6 mice per group). Group 1: Mice have injected with 20 μg HIV DNA vaccine and 30 ng 2D NSs. Group 2: Mice are injected with a 20 μg HIV DNA vaccine. Group 3: Mice are injected with 30 ng 2D NSs. Group 4: Mice are injected with PBS. Mice received three intramuscular injections. The spacing interval between two injections is three weeks. Mice are sacrificed at the 8 weeks. Mouse spleens and blood are harvested for flow cytometric assay and ELISA.

Flow cytometry

Freshly harvested mouse splenocytes are washed twice with PBS (3% fetal calf serum/ FBS, Gibco). Splenocytes with a concentration of 1×10^6 cells/mL are stimulated with 100 μl HIV epitope peptides (2 μg/mL) overnight at 37°C and 5% CO_2. Dimethylsulfoxide/DMSO (2 μL per well, Sigma) and staphylococcal enterotoxin B/SEB (1 μg/mL, Sigma) are negative and positive stimulators. Brefeldin A/BFA (1μg/ml,

Sigma) and monensin (1μg/ml, Sigma) are used for stopping the transportation of intracellular cytokines. After such a stimulation, splenocytes are stained with anti-mouse surface marker antibodies (Anti-Mouse CD3e FITC, Anti-Mouse CD8a Alexa Fluor® 700, Anti-Mouse CD4 APC-eFluor® 780, Anti-Mouse CD11b (LAMP-1) PerCP-eFluor® 710, CD11c PE) for 30 min at 4°C. After fixing with 2% paraformaldehyde (Sigma) for 15 min at 4°C, splenocytes are washed twice with PBS (3% FBS, Gibco) and are stained with monoclonal antibodies against intracellular targets (Anti-Mouse IFN gamma APC) for 30 min at 4°C (0.2% saponin for permeabilization). Splenocytes are washed twice with PBS and are analyzed on a FACS Calibur flow cytometer (Becton Dickinson). At least one million cells are gated for the analysis.

ELISA

96-well plates (Costar, Corning, NY) are coated with 0.01 μg/mL purified HIV Env proteins (gp145) at 4°C overnight. The coating buffer contains 0.012 mol/L Na_2CO_3 and 0.038 mol/L $NaHCO_3$ (pH 9.6). After washing five times with PBS, 96-well plates are blocked with PBS (3% BSA) at 37°C for two hours. Mouse serum samples are diluted with PBS (3% BSA) and added 100 μL into each well. After incubation at 37°C for one hour, plates are washed five times with PBS and incubated with 1:5000 diluted HRP-labeled antibodies against mouse IgG, IgG1, IgG2a, IgG2b or IgG3 (Santa Cruz Biotechnology) at 37°C for one hour. After washing five times with PBS, 100 μL freshly-prepared TMB substrate solution (Sigma, St. Louis, MO) is added to each well and incubated for 5 minutes. The reaction is stopped by 25 μL 2 M H_2SO_4. The optical density (OD) is detected at 630 nm or 450 nm by a Multiscan enzyme-linked

immunosorbent assay plate reader (Thermo Life Sciences, Hampshire, United Kingdom). The positive value is determined: (1) OD value (△value between 450 nm and 630 nm) is more than 0.1 at a dilution of 1:100; (2) OD value (△value between 450 nm and 630 nm) is at least 2.1 fold than of blank control. The serum samples from mice vaccinated with empty vector are used as blank control.

ELISPOT

ELISPOT is performed using a commercial kit from BD Pharmingen (Mouse IFN-γ ELISPOT Set). The cytokine capture antibody is prepared in the sterile PBS and coated on a PVDF plate overnight at 4°C. The plate is blocked with 3% BSA solution for 2 hours at room temperature. 1×10^5 fresh mouse splenocytes are seeded into the plate and stimulated with HIV Env peptides (5 μg/mL). The plate is incubated for 24 hours at 37°C and 5% CO_2. After washing four times with PBS, the plate is incubated for 2 hours with a biotinylated anti-mouse IFN-γ monoclonal antibody. The plate is incubated with an avidin horseradish peroxidase complex for 1 hour. After washing four times with PBS, the plate is incubated with peroxidase substrate AEC for 30 minutes. ELISPOT data detected with an automated ELISPOT reader system (Bio-Rad). More than 20 countings in 1×10^6 splenocytes is considered positive.

Protein-protein interaction (PPI) network construction

To screen crucial genes associated with 2D NSs-regulated HIV vaccine-triggered immune responses, differently expressed genes (DEGs) are mapped onto PPI data

collected from the Search Tool for the Retrieval of Interacting Genes (STRING) 10.0 database (https://string-db.org/). Interactions of DEGs with a confidence score ≥ 0.4 are selected for PPI network construction. PPI networks are visualized using Cytoscape software (3.6.0). Genes without interaction among other DEGs are removed in a network.

The analysis of cell viability

HeLa and HUVEC cells are cultured at 1×10^4 cells/well in a 96-well plate for 24 hours. A fresh cell culture medium containing 2D NSs with a series of concentrations (from 0 to 300 μg/mL) is added into each well (100 μL/well). After an incubation of 24 hours, cell viability is detected by a commercial CCK-8 kit.

***In vivo* distribution and toxicity analysis of 2D NSs**

Bal B/C mice of 8 weeks old are cultured in a pathogen-free environment. After injecting 2D NSs (10 mg/ Kg body weight) *via* tail vein, organs (liver, kidney, spleen, heart, lung) and serum samples of mice are harvested at different time points (the 1, 3, 5, 10 and 15 day). Er, and Dy ions in these organs are quantified using a ICP-MS. Organs are digested using acid (HNO_3:H_2O_2). The digest solution is diluted with 2% HNO_3 before subjecting to ICP-MS analysis. Organs from normal mice are used as control. Each sample is tested three times. Four biomarkers (alanine aminotransferase/ALT, aspartate aminotransferase/AST, creatinine/CREA, blood urea nitrogen/BUN) in serum are quantified using an automatic biochemical analyzer (Roche) at the 5, 10 and 15-day post

the injection of 30 ng 2D NSs. H&E-stained immunohistochemical slices from organs (liver, kidney, spleen, heart, lung) are observed using the optical microscope.

Statistical analysis

Values are shown as means ± standard deviations (SD). Analysis of differences among groups is conducted by one-way analysis of variance (ANOVA); $P<0.05$ is considered significant.

Supporting Figures

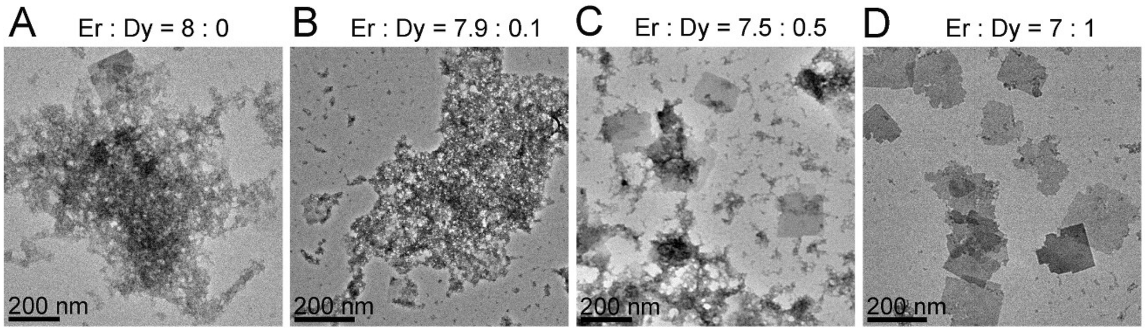

Figure S1. TEM images of 2D NSs with different ratios between Er and Dy. Effect of metal precursor in formation of sheet like. When the metal precursor ratio 7 (Er): 1 (Dy) produced complete sheet like morphology with limited contaminates.

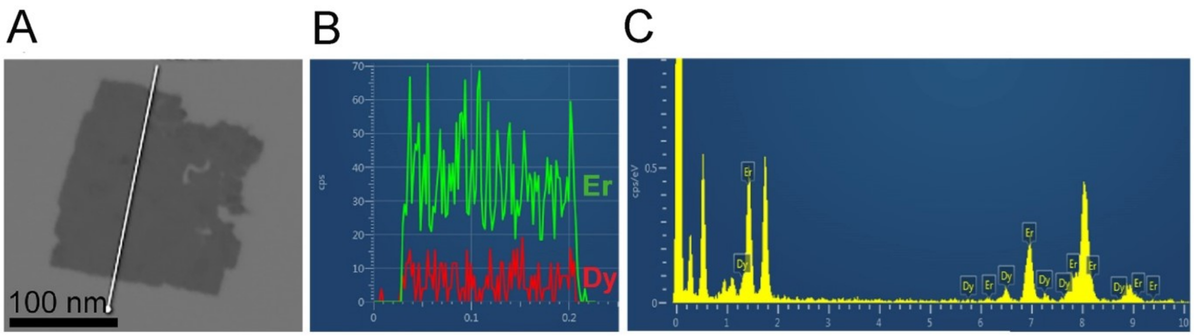

Figure S2. Chemical composition of 2D NSs. Line EDS spectra of single 2D NSs (A) line is drawn on a 2D NSs. (B) Collection of element Er and Dy along the line is recorded on a 2D NS. (C) EDS spectra collected along the line shows the presence of element Er and Dy in a 2D NSs.

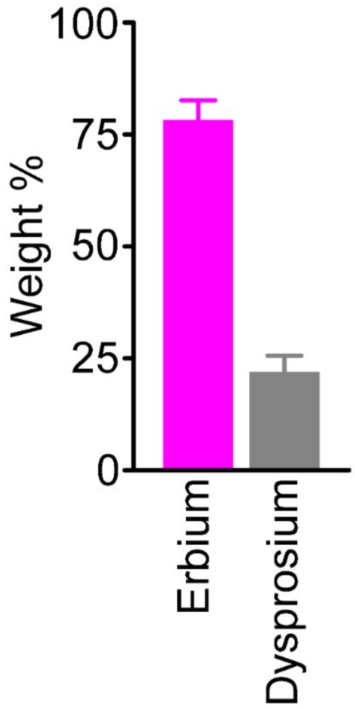

Figure S3. ICP-MS measurement of Er and Dy element distribution in NSs. Percentage distribution of element Er and Dy in the NSs.

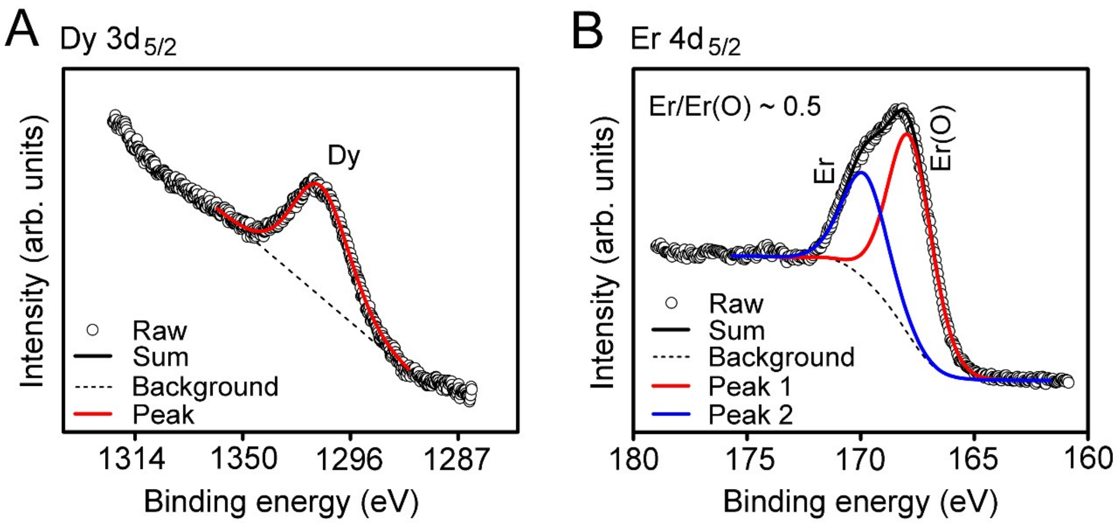

Figure S4. XPS fine structure measurement of ErDy NSs. High-resolution narrow scan XPS spectra of the 2D NSs measured in the A) Dy $3d_{5/2}$ and B) Er $4d_{5/2}$ binding energy regions.

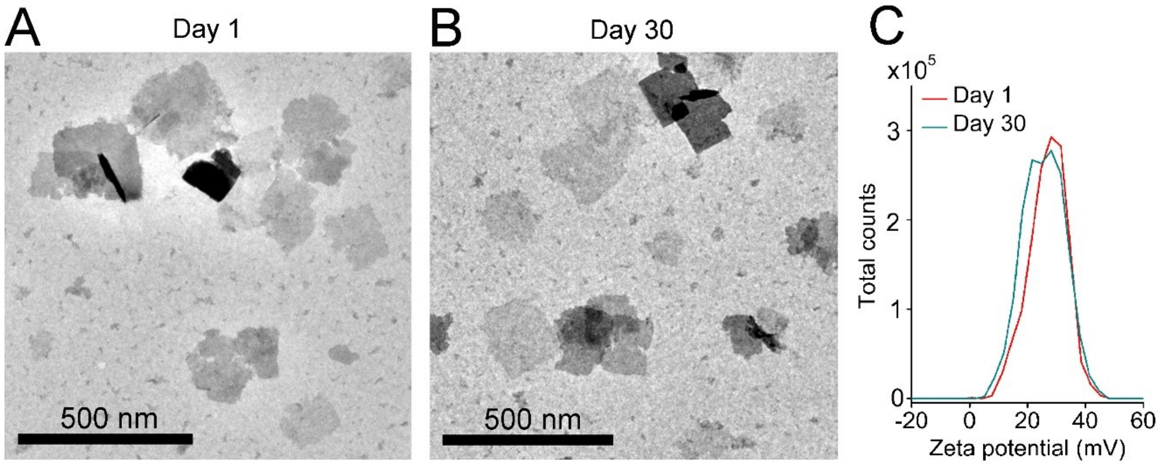

Figure S5. The stability of 2D NSs in water. The stability of 2D NSs in water is checked with TEM and zeta potential at day 1 and day 30 of synthesis. TEM shows the no significant change in morphology or disparity of 2D NSs at (A) Day1 and (B) at Day30. (C) Zeta plot shows almost similar zeta value of 2D NSs (Day 1 is +27.4 mV, Day 30 is + 26.3 mV).

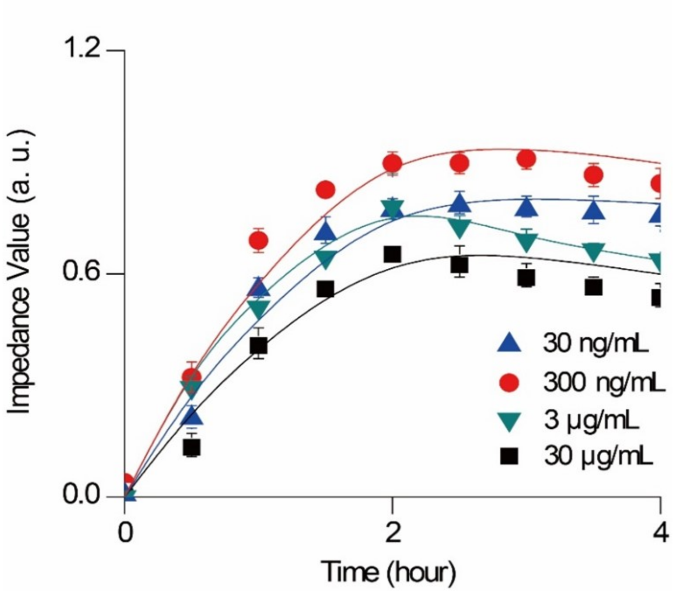

Figure S6. Electrical resistivity of macrophage exposed to 2D NSs. The change of electrical resistivity of macrophage-cultured plate during 4 hours. In comparison to normal macrophages, activated macrophages extend their size and cover broader area of plate, therefore resulting an increased electrical resistivity. Macrophages are stimulated with 30 ng/mL, 300 ng/mL, 3 μg/mL, and 30 μg/mL 2D NSs.

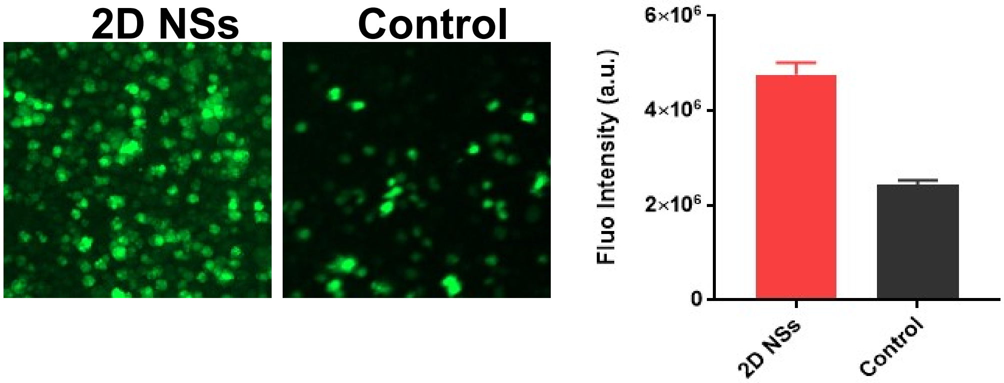
2D NSs
Control
6×10^6
4×10^6
2×10^6
0
Fluo Intensity (a.u.)
2D NSs
Control

Figure S7. The generation of ROS induced by 2D NSs. Macrophages are cultured in 24-well plates with a cell density of 5×10^5 cells/mL. 300 ng/mL 2D nanosheets are co-cultured with the cells for 24 h. Macrophages cultured into naked medium are reserved as blank control. Green fluorescent DCFH-DA dye is used as ROS probe. The cells are incubated at 37°C for 20 min and washed with serum-free cell culture solution three times. We observed green fluorescence (Left part), and quantified the intensity of green fluorescence (Left part) using a fluorimeter (BioTek fluorimeter) at the excitation wavelength of 488 nm and emission wavelength of 525 nm.

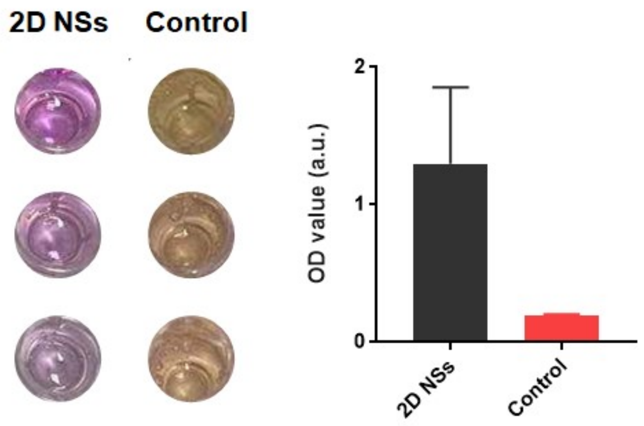

Figure S8. The generation of NO induced by 2D NSs. Macrophages are cultured in 24-well plates with a cell density of 5×10^5 cells/mL. 300 ng/mL 2D nanosheets are co-cultured with the cells for 24 h. Macrophages cultured into naked medium are reserved as blank control. We used SorLARBio NO Test Kit to detect the production of NO. We observed the production of NO by naked eyes (Left part), and quantified the concentration of NO by measuring the absorbance value at 550 nm.

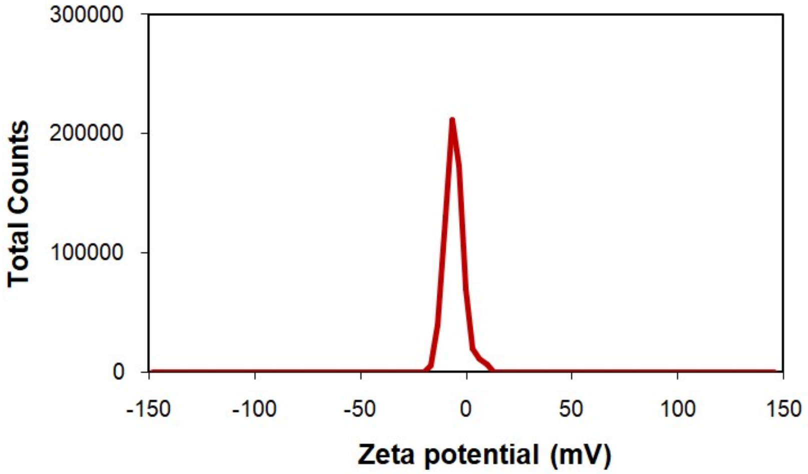

Figure S9. The zeta potential of HIV DNA-nanosheet complex (around -8 mV).

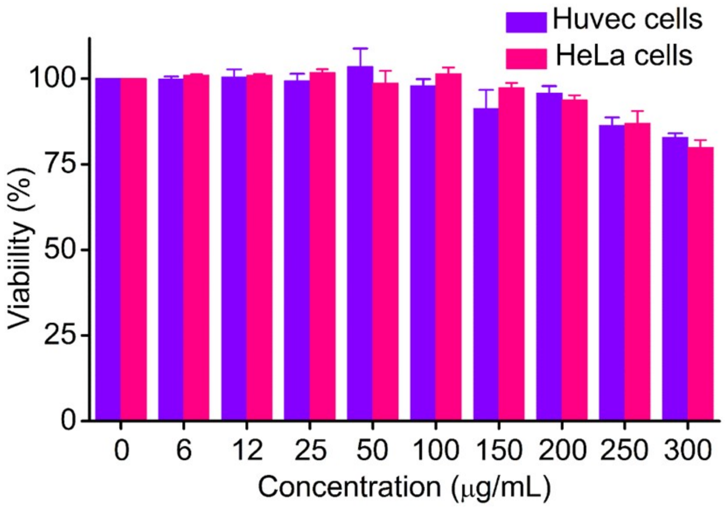

Figure S10. *In vitro* biocompatibility of 2D NSs. The viability of HUVEC and HeLa cells after exposing to 2D NSs with a series of concentration (from 0 to 300 μg/mL) for 24 hours.

Supporting Table

Table S1. Green fluorescent microspheres (2 μm size) are added into a cell culture well containing 1×10^6 macrophages. At Day 2, 4, 6, and 8, the fluorescence intensity of macrophages stimulated with either 300 ng/mL 2D NSs or 300 ng/mL LPS is detected *via* fluorescence confocal microscope. Normal macrophages cultured with green fluorescent microspheres (2 μm size) are used as control. The fluorescence intensity is shown as the mean value ± SD.

	**Fluorescence Intensity (a. u.)**			
	**Day 2**	**Day 4**	**Day 6**	**Day 8**
Naked macrophages	3.5 ± 2.79	8.15 ± 2.97	11.5 ± 2.98	13.66 ± 2.0
2D NSs + macrophages	11.59 ± 1.32	16.47 ± 3.77	11.5 ± 2.98	27.74 ± 3.08

LPS + macrophages	9.21 ± 1.72	13.39 ± 2.99	16.11 ± 3.16	23.09 ± 3.27
